# Fluid management in acute lung injury and ards

**DOI:** 10.1186/2110-5820-1-16

**Published:** 2011-05-30

**Authors:** Antoine Roch, Christophe Guervilly, Laurent Papazian

**Affiliations:** 1 Réanimation - Détresses Respiratoires et Infections Sévères, Hôpital Nord, Marseille 13015, France

## Abstract

ARDS is particularly characterized by pulmonary edema caused by an increase in pulmonary capillary permeability. It is considered that limiting pulmonary edema or accelerating its resorption through the modulation of fluid intake or oncotic pressure could be beneficial. This review discusses the principal clinical studies that have made it possible to progress in the optimization of the fluid state during ARDS. Notably, a randomized, multicenter study has suggested that fluid management with the goal to obtain zero fluid balance in ARDS patients without shock or renal failure significantly increases the number of days without mechanical ventilation. On the other hand, it is accepted that patients with hemodynamic failure must undergo early and adapted vascular filling. Liberal and conservative filling strategies are therefore complementary and should ideally follow each other in time in the same patient whose hemodynamic state progressively stabilizes. At present, although albumin treatment has been suggested to improve oxygenation transiently in ARDS patients, no sufficient evidence justifies its use to mitigate pulmonary edema and reduce respiratory morbidity. Finally, the resorption of alveolar edema occurs through an active mechanism, which can be pharmacologically upregluated. In this sense, the use of beta-2 agonists may be beneficial but further studies are needed to confirm preliminary promising results.

## Introduction

The ventilatory treatment of acute respiratory distress syndrome (ARDS) has greatly improved in recent years. During the same period, numerous nonventilatory therapies have been evaluated. Among them, modulation of fluid status and plasmatic oncotic pressure in patients have been the objects of studies--some promising, others disappointing in their physiological effects and outcome. ARDS is particularly characterized by pulmonary edema caused by an increase in pulmonary capillary permeability. In the early phase of ARDS, an associated septic state is usually responsible for hypovolemia. At this stage, hemodynamic optimization by early and adapted fluid loading has proven its prognostic value [1] and a fluid restriction strategy can result in hemodynamic aggravation and dysfunctions of associated organs, determining the mortality of patients with ARDS [2]. Subsequently, hemodynamic stabilization is generally associated with a resumption of diuresis and a decrease in body weight. Passage from one phase to another often is complex and difficult to distinguish but it is probably by identifying the transition between these two phases that one can detect the moment when a strategy of optimization of fluid balance on the restrictive side is possible. After a reminder on the physiopathologic bases, this review will present the principal clinical studies that have made possible to progress in the optimization of the fluid status during ARDS.

## The Consequences of Pulmonary Edema During ARDS

Even if pulmonary edema is only, in certain aspects, the reflection of the extent of alveolocapillary barrier lesions, it nevertheless has an impact on respiratory function at several levels [3]. An increase in pulmonary water triggers an early reduction in pulmonary compliance, which is responsible for an increase in respiratory work. At the alveolar edema stage, the intrapulmonary shunt is responsible for hypoxemia. The edema interacts with mechanical ventilation to facilitate pulmonary inflammation by rendering the lung heterogeneous on a ventilatory level, altering the surfactant and worsening alveolocapillary barrier lesions. Finally, edema is one of the principal determinants of pulmonary arterial hypertension, not only due to hypoxemia but also due to the pulmonary vascular compression that it triggers.

Thus, any attempt to reduce edema can potentially have beneficial effects on respiratory function and eventually outcome. However, the data that point to the prognostic role of the quantity of edema fluid in patients with ARDS or at risk of ARDS are very limited. In 1987, Simmons et al. observed that the evolution of body weight and fluid balance in ARDS patients [4] was correlated with outcome but without establishing a cause and effect relationship between them. Similarly, the prognostic role of the quantity of lung water has been suggested. Recently, Sakka et al. [5] retrospectively analyzed 373 intensive care unit (ICU) patients and found that the maximum quantity of lung water measured was a predictive factor for outcome. However, the prognostic effect of the quantity of edema fluid, measured by thermodilution, was not found in patients presenting ARDS in this study [5]. Conversely, lung water amount indexed with predicted body weight measured on day 1 of ARDS was found to be predictive of death in another study [6]. By definition, ARDS patients have severe pulmonary edema in the region of 15 to 20 ml.kg^-1 ^of body weight [7,8] and it is probable that other factors, extrapulmonary, will have a more marked prognostic impact. It should therefore be remembered that the degree of hypoxemia is a controversial prognostic factor during ARDS [9,10].

All in all, it makes sense to consider that limiting pulmonary edema or accelerating its resorption could be beneficial. However, even the prognostic role of the quantity of pulmonary edema fluid remains uncertain.

## ARDS: Lesional or Hemodynamic Edema?

Under physiologic conditions, there is a transfer of fluid from the capillary lumina toward the interstitium. In fact, on the one hand, the endothelium presents some permeability, the consequences of the forces acting on both sides of the endothelium favor an extravasation of fluid [11]. Fluid flux through the pulmonary endothelium is quite correctly estimated by Starling's equation, which expresses the fact that net filtration flux is the product of the hydraulic conductance of the exchange surface barrier by effective filtration pressure. The equation is expressed as follows: Jv = K [(Pc - Pi) - S (πc-πi)], where Jv is the flow of fluid through the capillary wall, K is the capillary hydraulic filtration coefficient reflecting endothelial water permeability, Pc is capillary hydrostatic pressure, Pi is interstitial hydrostatic pressure, S is the oncotic reflection coefficient, πc is the capillary oncotic pressure, and πi is the interstitial oncotic pressure. The first part of the equation represents the hydrostatic pressure gradient, which tends to produce a flow of fluid out of the vessels. The second part represents the oncotic pressure gradient, which opposes this transudation of fluid. The influence of oncotic pressure on fluid flux through the endothelium is modulated by the oncotic reflection coefficient (S), which represents the permeability of the endothelium to oncotically active substances.

Schematically, two principal mechanisms can work toward an increase in pulmonary water and possibly alveolar flooding: on the one hand, an increase in pulmonary microvascular pressure and on the other hand, an increase in alveolocapillary barrier permeability. During ARDS, an increase in endothelial permeability is a fundamental element in the formation of pulmonary edema [12]. On a hydrodynamic level, the capillary hydraulic filtration coefficient increases and the reflection coefficient of oncotically active substances diminishes or even goes to nearly zero in certain zones. Thus, the oncotic pressure gradient, which normally opposes the formation of edema, is little or no longer effective. Consequently, a given augmentation in hydrostatic pressure will trigger a greater augmentation in fluid efflux if alveolocapillary barrier permeability is increased. This has been well illustrated in experimental studies by Guyton [13], showing that edema forms faster and at a lower hydrostatic pressure threshold when one has first damaged the alveolocapillary barrier before progressively increasing the left atrium pressure. Moreover, other experimental studies have shown that even a modest decrease in pulmonary capillary pressure by vasodilatation or early reduction of volemia in a lesional edema model can limit the formation of pulmonary edema [14-16].

Clinically, even if the presence of pulmonary artery occlusion pressure (PAOP) inferior to 18 mmHg has been defined for a diagnosis of increased pulmonary permeability edema, a potential role of hydrostatic forces is not excluded in ARDS. First, PAOP underestimates capillary filtration pressure, especially in ARDS where the pulmonary venous resistance is increased [17,18]. Second, in case of increased alveolocapillary permeability, the critical hydrostatic pressure above which pulmonary edema will develop will be lower than in the case of normal lung permeability. If the capillary hydraulic filtration coefficient is doubled, critical hydrostatic pressure will only be 10 mmHg. The notion that there is no normal capillary pressure in case of permeability edema constitutes a theoretical justification for limiting pulmonary filtration pressure in case of increased permeability pulmonary edema or in a patient at risk and therefore for measuring or estimating capillary pressure which is a fairly true reflection. Most clinical studies have shown that the majority of patients with ARDS have a PAOP that is superior to critical filtration pressure values when the barrier is damaged (Table 1). Among the 1,000 patients enrolled in a recent randomized, controlled study [19], 29% had a PAOP above 18 mmHg while cardiac index was normal or high, suggesting that high filling pressures observed were not the result of congestive heart failure. Knowing that the PAOP is inferior to capillary hydrostatic pressure, one can suppose that these patients more often will have capillary pressure that is beyond critical pressure.

**Table 1:** Mean values for mean pulmonary arterial pressure (MPAP), pulmonary capillary pressure (PCP), and pulmonary artery occlusion pressure (PAOP) in several clinical studies that studied these parameters in ARDS patients.

n	MPAP	PCP	PAOP	Reference number
1,000	na	na	15.6 ± 0.4	[19]
10	28 ± 1	17 ± 1	13 ± 1	[22]
18	34 ± 2	25 ± 1	17 ± 1	[23]
7	27 ± 3	15 ± 1	10 ± 1	[24]
15	40 ± 2	27 ± 2	22 ± 1	[46]
8	39 ± 2	28 ± 2	15 ± 1	[47]

na = not available.

One can understand the potential interest in limiting pulmonary microvascular pressure during the period that endothelial permeability is increased. However, this is difficult to consider in practice for several reasons. The first is that in the early phase of inflammatory pulmonary aggression when the edema is developing, therapeutic strategy is usually oriented toward systemic hemodynamic recovery, associating volume resuscitation and vasopressors with the quite contradictory goal to reduce pulmonary capillary pressure. The second reason is the difficulty of measuring or evaluating pulmonary capillary filtration pressure. Some factors, such as vasoplegia and hypovolemia, combine to diminish pulmonary capillary pressure, whereas the local production of vasoconstrictive mediators or myocardial depression can increase it. In addition, effective filtration pressure is the resulting complex of venous, capillar and arteriolar pressures, all three of which can be affected by vasomotor mediators with contradictory effects.

All in all, maintaining a low pulmonary capillary pressure is understandable, especially early, during the phase where permeability is increased. However, considering it as a therapeutic objective is difficult, because correction of hypovolemia is mandatory and measuring the true capillary pressure is difficult.

## Resorption and Drainage of Pulmonary Edema

As we have seen, ARDS is particularly characterized by pulmonary edema. The part of the interstitial water that is in excess and that will not flood the alveoli is drained by the lymphatic network. The fluid that is not drained by the lymphatic network--because it is saturated--accumulates in the loose peri-bronchovascular conjunctive tissue of the hila, which is the first accumulation site in pulmonary edema. These zones have at the same time very low resistance to fluid flux and major compliance. Thus, because interstitial pressure remains low, resorption of interstitial edema is little or not at all produced by pulmonary vascular resorption with the pressure remaining in favor of transudation of the vascular sector toward the interstitium. On the other hand, interstitial drainage will depend on the capacity of lymphatic flow to increase as well as the capacity of the perihilar tubes to drain into the mediastinum and the interstitial edema to evacuate toward the pleura and then the lymphatic system. Edema drainage will therefore be increased if right atrial pressure decreases and if pleural pressure is low.

Once in the alveoli, the water is absorbed from the alveoli toward the interstitium by active transepithelial ionic migration, which creates an osmotic gradient leading to water reabsorption toward the interstitium [20]. This water then goes into the interstitial edema drainage circuit. Thus, alveolar edema resorption is not performed by communicating vessels with pulmonary circulation. During ARDS, active transport of ions and fluid by the epithelium is altered due to rupture of the alveolocapillary barrier and epithelial cell dysfunction. Water reabsorption requires that the fluid leak first be reduced not only by reduction of endothelial permeability but also possibly by modifications in epithelial cell form, making the epithelium more solidly impermeable. Alveolar fluid clearance is altered in most patients with ARDS, so maintenance of normal clearance or its increase is reported to be associated with better outcome [21].

Overall, one can theoretically improve interstitial pulmonary edema clearance and facilitate edema prevention mechanisms by diminishing both central venous pressure and pleural pressure. Parallel stimulation of alveolar edema resorption toward the interstitium could be complementary.

## Fluid Restriction and Diuretics: Clinical Studies and Practical Consequences

From what we have seen above, even if it is possible that limitation of pulmonary capillary pressure and facilitation of edema drainage by fluid restriction could limit the amount of lung edema or facilitate its elimination, it is not known if there is a clinical interest in diminishing pulmonary edema. We therefore will see that the current data in the literature does not make it possible to establish that the beneficial effects of fluid restriction have an effect on pulmonary function.

In addition to ventilatory or inotropic therapies, one can theoretically reduce capillary pressure in two manners: by diminishing the volemia or by vasodilitating the pulmonary vessels. Selective pulmonary vasodilatation, in particular by inhaled nitric oxide (NO) or prostaglandins, has been the object of experimental studies in increased permeability pulmonary edema models. Some have reported that a reduction in pulmonary water or endothelial permeability could be connected to a decrease in capillary filtration pressure [16]. However, most of the clinical studies in ARDS patients have only shown modest effects by these drugs on pulmonary capillary pressure [22-24], and the most recent large studies have not reported prognostic benefit with the systematic use of inhaled NO in cases of acute lung injury or ARDS [25].

Fluid restriction that is more or less associated with a diuretic treatment makes it possible to reduce both pulmonary capillary pressure and central venous pressure. The problem with fluid restriction is that it often is difficult to perform because of the often precarious hemodynamic state of such patients. Until recently, only a few prospective studies had been undertaken and have suggested a reduction in respiratory morbidity with fluid restriction. In 1990, Humphrey et al. [26] suggested that interventional reduction of PAOP in ARDS patients improved mortality. However, the study presented numerous limitations. In a very limited population studied retrospectively, the authors showed that in patients whose PAOP could be reduced by 25%, mortality was lower than in those whose PAOP could not be lowered. No link between PAOP reduction and outcome could be established. In 1992, Mitchell et al. [8] performed a randomized study of 101 ICU patients. In 52 patients, hydration was based on the measurement of extravascular lung water by double dilution and 49 patients were monitored by pulmonary arterial catheter and PAOP. In the group monitored by extravascular lung water measurement, hydration strategy was based on a restriction of fluid intake associated with the use of vasopressors if extravascular lung was superior to 7 ml.kg^-1 ^or on preferential filling in case of low extravascular lung water, whereas in the other group, the PAOP objective was 10 mmHg if the hemodynamic state was normal and 18 mmHg in case of hypotension. In patients with higher PAOP, they used vasopressors or vasodilatators depending on blood pressure. Fluid loading was used in patients with lower PAOP. The strategy based on lowering extravascular lung water resulted in a shorter duration of both ventilation and ICU stay than the PAOP strategy. The cumulated fluid result for the first 3 days was + 2 liters in the "PAOP" group, whereas it was zero in the "extravascular lung water" group in which PAOP also significantly diminished. Extravascular lung water diminished by 25% in the "extravascular lung water" group, whereas it did not change in the other group, suggesting that it was really fluid restriction that acted on extravascular lung water reduction and that it influenced outcome. There was no increase in vasopressor requirements.

More recently, a randomized, multicenter study evaluated a strategy of fluid restriction that was more or less associated with diuretic treatment prescribed in the absence of hypotension and renal failure in patients with acute lung injury or ARDS [19]. Patients were included in the study approximately 48 hours after admission to the ICU. The decision to submit patients to fluid loading or to diuretic treatment depended on the presence of oliguria and the level of central venous pressure (CVP) of PAOP. Schematically, the goal was to obtain a CVP of 8 mmHg or less in the "conservative-strategy" group or 14 mmHg in the "liberal-strategy" group. In the patients monitored by pulmonary arterial catheter, the objectives of PAOP were 12 mmHg in the conservative-strategy group and 18 mmHg in the liberal-strategy group. The protocol was applied for 7 days after inclusion of the patient but was not applied in cases of hypotension. The conservative strategy resulted in zero fluid in 7 days, whereas the fluid results in the liberal-strategy group were +6 liters over 7 days. The conservative strategy discreetly improved oxygenation in patients and increased the number of days without ventilation (14.6 ± 0.5 vs. 12 ± 0.5; *p *< 0.001) but did not influence mortality at 60 days, which was the principal goal of the study. This study confirmed the impression that limiting the fluid intake of patients with isolated respiratory failure can limit respiratory morbidity without aggravating other organ dysfunctions. However, the exclusion of hemodynamically unstable patients or patients with renal failure makes it impossible to generalize these results or to create a "gold standard" for management of the fluid status of every ARDS patient. In addition, the absence of an effect on mortality reminds us that the management of fluid intake in ICU patients does not boil down to being liberal or conservative. In most patients, ARDS is described within the framework of an early and generalized systemic inflammation that is responsible for hemodynamic dysfunction. At this stage, hemodynamic restoration based on early fluid administration constitutes one of the cornerstones for improving outcome [1]. It is only once the initial phase of instability has passed that a reasonable policy of fluid intake aimed at zero fluid can contribute to reducing the duration of ventilation and ICU stay [27]. The importance of a "biphase" fluid strategy was recently illustrated by a retrospective study that included 212 patients presenting acute lung injury (ALI) complicating septic shock [28]. In this study, the non-performance of early adapted fluid administration and the absence of a negative fluid balance during a minimum of the first 2 consecutive days within the 7 days following the occurrence of septic shock were independent mortality factors in multivariate analysis. Along these lines, the Surviving Sepsis Campaign [29] recommends a conservative fluid strategy in patients with ARDS or ALI and in the absence of shock. Other recent studies have stressed the influence of fluid balance on outcome [30,31]. However, their design does not make it possible to confirm if an interventional strategy, such as that of the FACTT study [19], influences the outcome of ARDS patients. According to the literature, the beneficial effect of conservative therapy also could come from extrarespiratory mechanisms. Thus, the patients treated with a conservative strategy in the FACTT study [19] had better neurological status - perhaps because the patients were desedated earlier because of better respiratory status, perhaps because they had less severe cerebral edema. Moreover, the patients with conservative treatment received fewer transfusions whose potentially deleterious role is well known in intensive care [32]. A simplified version of the algorithm used in the FACTT study was published by the ARDS Network and discussed in a recent review on the subject [33].

## Modulation of Oncotic or Osmotic Pressure: The Effects of Administering Albumin or Hypertonic Saline

From a hemodynamic point of view, in case of a decrease in plasmatic oncotic pressure, which is clinically illustrated by hypoprotidemia/hypoalbuminemia, pulmonary edema forms at a lower hydrostatic pressure because the oncotic pressure gradient between plasma and the interstitium decreases. Therefore, experimentally, whereas the pulmonary edema begins to develop at a pressure of 24 mmHg when oncotic pressure is normal, it begins at 11 mmHg when it is reduced [13]. Hypoprotidemia therefore facilitates the development of hydrostatic pulmonary edema. This is potentially important in ICU patients in whom hemodiluation and catabolism associate to diminish protidemia. However, the importance of oncotic pressure in the limitation of flux is only conceivable if the barrier is intact. In case of endothelial lesions, an interstitial edema will be all the richer in proteins than the plasma, theoretically limiting the interest of increasing the plasmatic oncotic pressure. Thus, in animal models, an increase in oncotic pressure during the early phase of a lesional edema will not limit the formation of edema [14].

On analyzing a cohort of 455 septic patients with a risk of ARDS, Mangialardi et al. [34] found that hypoprotidemia was an independent predictor of the occurrence of ARDS. Subsequently, the same team [35] evaluated the interest of a strategy of diuretic treatment associated with albumin filling in ARDS patients with a protidemia inferior to 50 g/l. Of the 37 patients included in the study, 19 received an association of albumin (75 g/d) and furosemide for 5 days and 18 received placebos. When the protidemia was superior to 60 g/l in the treated group, the treatments were replaced by placebos. Continuous infusion of furosemide was adjusted to obtain a weight loss of at least 1 kg/d without exceeding 8 mg/h of furosemide. The patients in the treated group presented a PaO_2_/FiO_2 _ratio that was slightly and transitionally better than that of the placebo group, without other beneficial or deleterious effects, particularly on renal function. In a second study [36] to distinguish the effects of albumin and diuretics, the same authors randomized 40 patients; 20 received furosemide alone and the other 20 received furosemide and albumin (75 g/d) for 3 days. When protidemia was superior to 80 g/l in the treated group, albumin was replaced with a placebo. Albuminemia increased by 13 g/l in the albumin + furosemide group, reaching 30 g/l at the end of treatment and increased by 3 g/l in the furosemide alone group, reaching 20 g/l at the end of treatment. Once again, the effects can be summarized by a discrete improvement in oxygenation when albumin was associated with diuretic treatment compared with diuretic treatment alone. A large randomized study [37] demonstrated that volume therapy using albumin was equivalent to volume therapy using saline in ICU patients. At this time, the very limited clinical data do not make it possible to recommend the administration of albumin with the goal to improve pulmonary function and respiratory morbidity in ARDS patients.

The use of hyperosmolar filling solutions, such as hypertonic saline, could have the advantage, due to the limited amount of fluid administered, of limiting the development of pulmonary edema in case of an increase in the alveolocapillary barrier. Moreover, the use of hypertonic solutions has limited pulmonary injury after hemorrhagic shock in experimental models by improving splanchnic output and reducing the adhesion and cytotoxicity of neutrophils compared with the use of isotonic solutions [38,39]. In clinical practice, the potential interest of hypertonic resuscitation has been investigated in patients with traumatic hypovolemic shock. In a randomized study that included 422 patients, evolution toward ALI was less frequent when patients had received fluid loading with hypertonic saline/dextran than with normal saline [40]. However, in a recent randomized study, including 853 patients, fluid loading with hypertonic saline or hypertonic saline/dextran neither reduced mortality nor prevented ARDS occurrence compared with normal saline resuscitation [41].

Roch et al. [42] compared in a porcine model of hemorrhagic shock the effect of isotonic and hypertonic solutions on the occurrence of pulmonary lesions and on inflammation according to precise hemodynamic criteria. The use of hypertonic saline did not exert a preventive effect on the appearance of ALI and pulmonary edema after hemorrhage in this study. Those authors even observed a deleterious effect with the prior use of hypertonic saline before performing experimental ischemia-reperfusion by clamping the pulmonary arteries [43]. This effect appeared to be independent of the hemodynamic effects of saline but was more probably linked to a direct effect on alveolocapillary barrier permeability.

## Increasing Resorption of Alveolar Edema - A Complementary Objective

This therapeutic aspect does not strictly imply manipulation of fluid balance in patients. However, it deserves to be mentioned because it clearly shows that resorption of alveolar edema does not occur by manipulation of vascular pressures, but rather by stimulation of active water transport from the alveoli toward interstitium, which would be complementary to strategies favoring the draining of interstitial edema.

Despite severe epithelial lesions, alveolar clearance is usually pharmacologically stimulable. Several experimental studies have shown that the exogenous administration of cAMP agonists, in particular beta-2 agonists, accelerates the resolution of edema, whatever its nature [44]. The action of beta-2 agonists principally occurs through an increase in the quantity and activity of Na/K pumps in the basal membrane and sodium canals in the pneumocyte apical membrane whose effect is to increase the sodium gradient between the alveoli and the interstitium and therefore the absorption of water. A recent clinical study [45] showed that the administration of IV salbutamol at a dose of 15 μg/kg/h for 7 d in ARDS patients made it possible to diminish the quantity of pulmonary water measured by transpulmonary thermodilution without affecting oxygenation, duration of mechanical ventilation, or outcome. However, it was a preliminary study that included only 40 patients.

## Conclusions

Fluid management with the goal to obtain zero fluid balance in ARDS patients without shock or renal failure significantly increases the number of days without mechanical ventilation [19]. On the other hand, patients with hemodynamic failure must receive early and adapted fluid resuscitation [1]. Liberal and conservative fluid strategies are therefore complementary and should ideally follow each other in time in the same patient whose hemodynamic state progressively stabilizes. At present, albumin treatment does not appear to be justified for limitation of pulmonary edema and respiratory morbidity. Finally, the resorption of alveolar edema occurs through an active mechanism, which can be pharmacologically upregluated. In this sense, the use of beta-2 agonists may be beneficial, but further studies are needed to confirm preliminary promising results.

## Competing interests

The authors declare that they have no competing interests.

## Authors' contributions

AR, CG, and LP drafted the manuscript. All authors read and approved the final manuscript.
